# First person – Martin Crivello and Marion Hogg

**DOI:** 10.1242/dmm.041731

**Published:** 2019-08-13

## Abstract

First Person is a series of interviews with the first authors of a selection of papers published in Disease Models & Mechanisms, helping early-career researchers promote themselves alongside their papers. Martin Crivello and Marion Hogg are co-first authors on ‘Vascular regression precedes motor neuron loss in the FUS (1-359) ALS mouse model’, published in DMM. Martin, who is interested in molecular biology and neuroscience, will soon be joining Eurofins in a scientist position. Marion is a senior postdoc in the lab of Prof. Jochen Prehn at the Department of Physiology & Medical Physics and SFI FutureNeuro Research Centre, Royal College of Surgeons in Ireland, Dublin, Ireland, investigating transfer RNA (tRNA) cleavage by the ALS-associated ribonuclease angiogenin and the fate and function of tRNA-derived fragments.


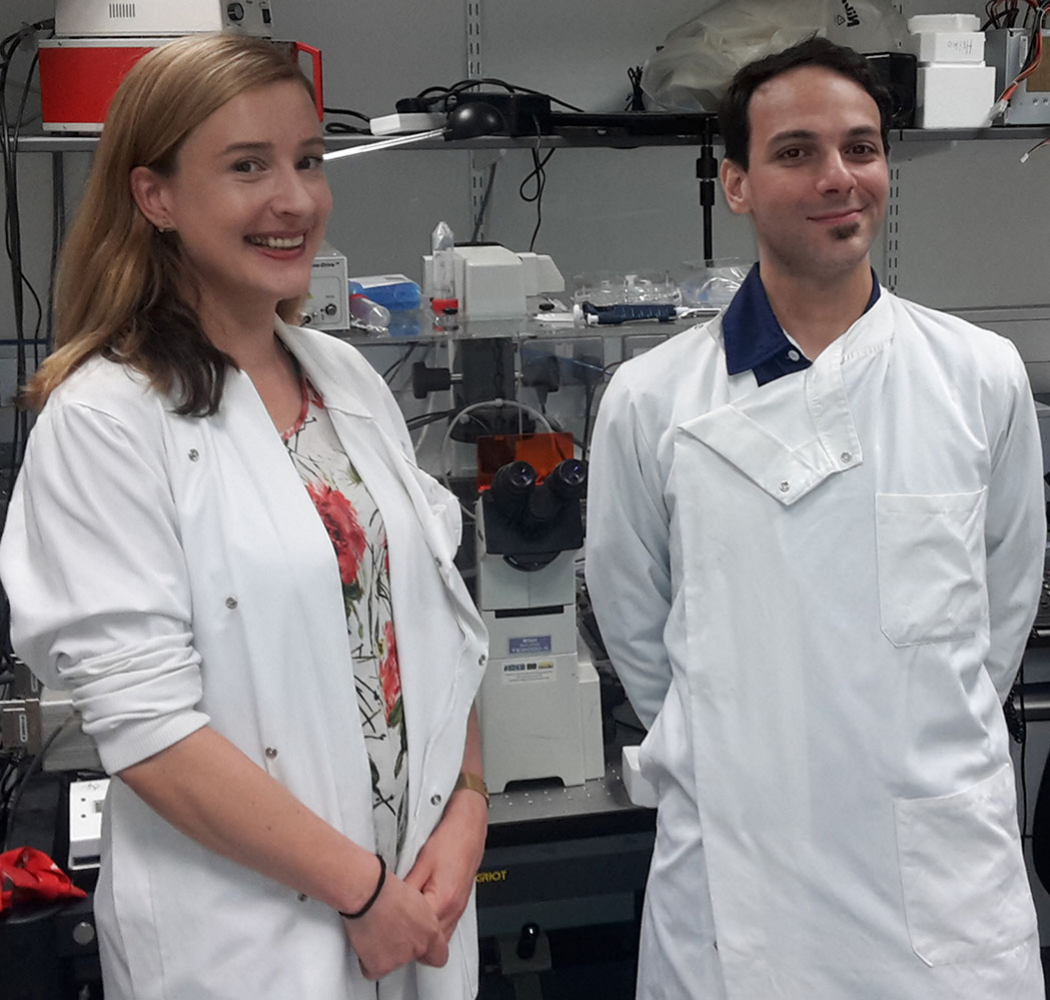


**Marion Hogg and Martin Crivello**

**How would you explain the main findings of your paper to non-scientific family and friends?**

MC+MH: Amyotrophic lateral sclerosis, or ALS, is a neurodegenerative disease that leads to paralysis and death; you may be familiar with it thanks to the ‘Ice Bucket Challenge’. Sadly, after decades of research, there are few treatment options available, and they are not very effective either. We previously showed that there are defects in the vascular system of the mutant SOD1 mouse model of ALS and that treatment with angiogenin corrected this defect and improved survival. Our research here was conducted on a mouse model that features a truncated version of a different gene, FUS (1-359), that is also known to be associated with the disease. We found vascular defects present before the loss of motor neurons (which control muscle movement). Sadly, FUS (1-359) mice did not respond to angiogenin treatment.

**What are the potential implications of these results for your field of research?**

MC: Obviously, seeing our treatment paradigm fail here was not the result I was hoping for, but this is still important. First, we did describe vascular and gene expression issues, which will hopefully prove useful for our lab or others to build upon. Even the negative treatment results can help focus different promising drugs on the more appropriate model or reveal key differences between different ALS subgroups.

“Even the negative treatment results can help […] reveal key differences between different ALS subgroups.” – *Martin Crivello*

MH: This study shows that similar disease processes occur in different ALS mouse models, highlighting them as good therapeutic targets. However, it also suggests that even when there are common pathways, therapeutics may not work in the same way because of subtle differences between different ALS mouse models. Conflicting results in the preclinical assessment of therapeutics in different transgenic mouse models highlights how ALS is a heterogeneous disorder. The requirement for therapeutics to show efficacy in multiple different models may limit the development of drugs that could be effective in a defined subset of patients.

**What are the main advantages and drawbacks of the model system you have used as it relates to the disease you are investigating?**

MC: I think the main advantage is that this is a relatively novel model and that it allows us to study a particular ALS subgroup. For example, the most widely used ALS mouse model features a mutation the SOD1 gene, but this is not necessarily representative of the whole ALS spectrum. One disadvantage is that the FUS (1-359) model we used presents a rapid decline, meaning you have to pay close attention to the mice daily to prevent inhumane levels of deterioration.

MH: The FUS (1-359) mice accurately model the symptoms of ALS, as transgenic mice from this colony have a very variable onset of symptoms and a very rapid disease progression (2 weeks from onset to death). This replicates what is seen in humans with ALS; however, the unpredictability of the model makes it very difficult for researchers to work with. The high variability in symptom onset is surprising given that these mice are on a genetically identical background and maintained in a constant environment.

**What has surprised you the most while conducting your research?**

MH: The newer ALS mouse models we have worked with [here, the FUS (1-359) model, and in other studies the TDP-43^A315T^ model] show a surprising amount of variation in symptom onset. Some transgenic mice from these models get sick at around 2 months of age, whereas others do not show symptoms until around 5 months of age or later, despite being maintained on inbred backgrounds and within the same environment. This accurately models what is seen in ALS patients and shows there is still a lot to learn about ALS heterogeneity.

**Figure:**
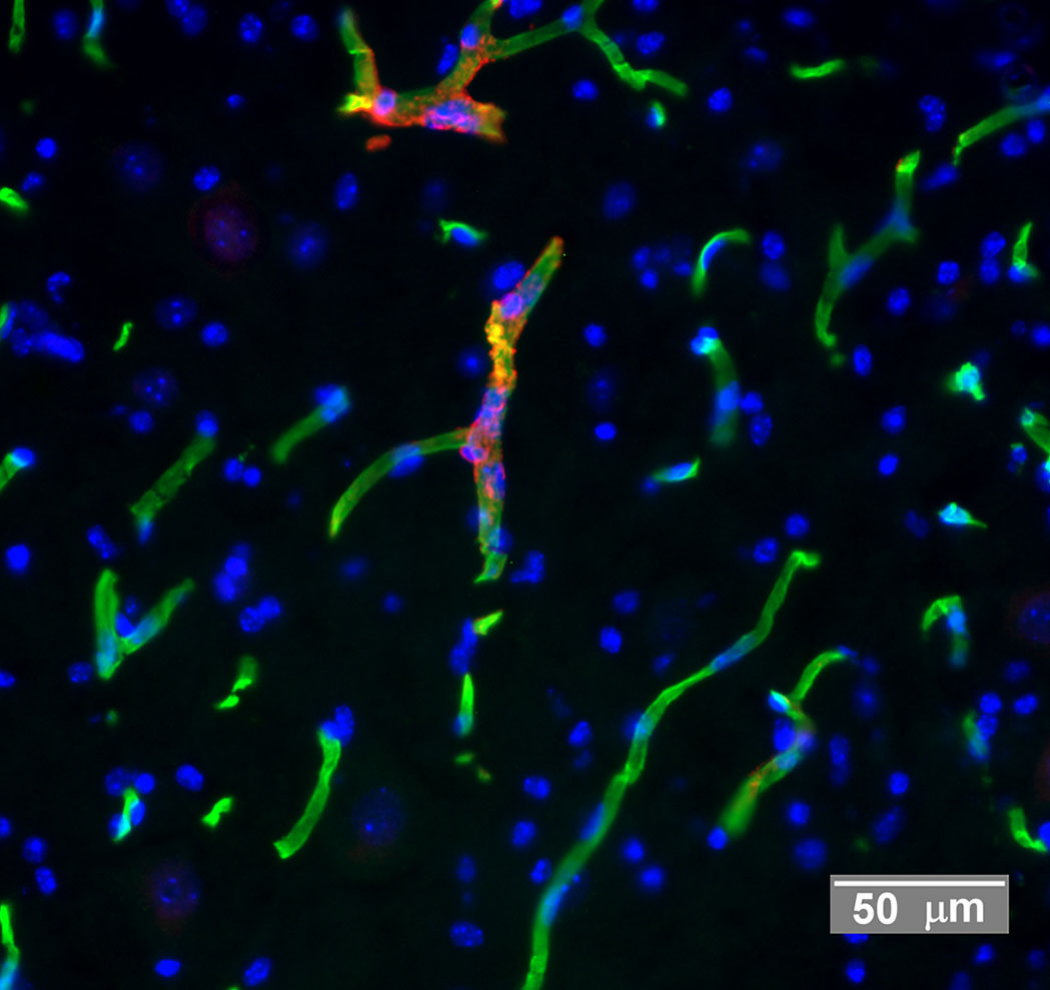
**False-colour microphotograph of mouse spinal cord tissue showing endothelial cells (green), pericytes (red) and cell nuclei (blue).**

**Describe what you think is the most significant challenge impacting your research at this time and how will this be addressed over the next 10 years?**

MC: With ALS there has been a long string of failures in translating promising drug research into the clinic. This may be partly because of how heterogeneous this disease can be and I'm hopeful that, as personalized medicine becomes more mainstream in the coming years, preclinical research and clinical trials will be better equipped to differentiate failed drugs from those useful only for a very specific subset of patients.

MH: ALS is a clinically heterogeneous disorder, which makes investigating disease mechanisms difficult. There may be multiple pathogenic processes occurring at the same time, some of which are specific to a subset of patients, and others that may be common to many. I think precision medicine will improve research into new treatments that may target some but not all of these processes. First, we need to develop methods to identify which processes are going wrong in which patients, and to do that we need to identify biomarkers that can indicate the underlying problem. Then we need to develop targeted treatments for each specific cause. Then there would be the potential for each patient to be prescribed a cocktail of treatments to address their specific needs.

“I think precision medicine will improve research into new treatments” – *Marion Hogg*

**What changes do you think could improve the professional lives of early-career scientists?**

MC: If you are working towards your PhD, pay is low, working hours are long and you are usually lacking in workers’ rights. Postdocs are in a better situation, but we still contend with job insecurity as we move from short-term contract to short-term contract. At this point, I think turning PhD ‘students’ into (better paid) fully recognized workers would ease off a lot of stress from those who make up the backbone of the scientific endeavour.

MH: More starter grants to help junior researchers like me to take the first step on the road to independence.

**What's next for you?**

MC: I will be soon starting an exciting role as a scientist at Eurofins, involving molecular biology work. I think this will present a lot of opportunities for my professional growth that may not be present in an academic setting.

MH: I hope to start my own independent research group soon investigating tRNA fragments in neurological disorders, including ALS.
